# High-Throughput Classification of Radiographs Using Deep Convolutional Neural Networks

**DOI:** 10.1007/s10278-016-9914-9

**Published:** 2016-10-11

**Authors:** Alvin Rajkomar, Sneha Lingam, Andrew G. Taylor, Michael Blum, John Mongan

**Affiliations:** 1 Department of Medicine, Division of Hospital Medicine, University of California, San Francisco, 533 Parnassus Ave., Suite 127a, San Francisco, CA 94143-0131 USA; 2 Center for Digital Health Innovation, University of California, San Francisco, San Francisco, CA USA; 3 Department of Radiology and Biomedical Imaging, University of California, San Francisco, San Francisco, CA USA

**Keywords:** Radiography, Chest radiographs, Machine learning, Artificial neural networks, Computer vision, Deep learning, Convolutional neural network

## Abstract

The study aimed to determine if computer vision techniques rooted in deep learning can use a small set of radiographs to perform clinically relevant image classification with high fidelity. One thousand eight hundred eighty-five chest radiographs on 909 patients obtained between January 2013 and July 2015 at our institution were retrieved and anonymized. The source images were manually annotated as frontal or lateral and randomly divided into training, validation, and test sets. Training and validation sets were augmented to over 150,000 images using standard image manipulations. We then pre-trained a series of deep convolutional networks based on the open-source GoogLeNet with various transformations of the open-source ImageNet (non-radiology) images. These trained networks were then fine-tuned using the original and augmented radiology images. The model with highest validation accuracy was applied to our institutional test set and a publicly available set. Accuracy was assessed by using the Youden Index to set a binary cutoff for frontal or lateral classification. This retrospective study was IRB approved prior to initiation. A network pre-trained on 1.2 million greyscale ImageNet images and fine-tuned on augmented radiographs was chosen. The binary classification method correctly classified 100 % (95 % CI 99.73–100 %) of both our test set and the publicly available images. Classification was rapid, at 38 images per second. A deep convolutional neural network created using non-radiological images, and an augmented set of radiographs is effective in highly accurate classification of chest radiograph view type and is a feasible, rapid method for high-throughput annotation.

## Background

A core task of diagnostic radiology is to identify pathologic abnormalities in high-resolution images that vary in appearance due to pathology, patient orientation, normal anatomic variation, prior medical interventions (e.g., pacemaker placement), or from differences in image capture techniques or machines. A component of initial image interpretation is performing a set of classification tasks to identify objects in an image, which machine learning is well suited for. This suggests that machine learning may be particularly helpful to assist in identification of features in radiographs [1]. However, despite decades of effort to help automate image interpretation [2, 3], relatively few algorithms for computer-aided diagnosis have been incorporated into widespread clinical use (breast imaging being a notable exception) [4].

Computer vision underwent a revolution in 2012, when deep learning approaches that harnessed convolutional neural networks halved the error rates in standardized image classification challenges compared to other best competitors [5, 6]. The new approach allowed machine learning systems to operate on raw data rather than manually created features or image segments [6, 7], learning multiple levels of abstract representation of an image. Once trained, it is optimized for fast and efficient classification of images. However, high-performing algorithms have hundreds of millions of parameters that must be learned from the data, requiring large numbers of images and efficient hardware implementations for effective training.

The application of deep learning techniques to radiological images is rapidly expanding [8–15], but one challenge in applying neural networks to radiological images is obtaining access to sufficient quantities of image data. It is often difficult for researchers to access images outside of their institution due to patient privacy issues. Although a picture archiving and communication system (PACS) for a single institution may contain several million studies, the number of studies of a particular type is much smaller, and it may not be feasible to obtain enough images to fully train a network. In contrast, non-radiological images are widely available in much greater numbers. Techniques that leverage the availability of non-radiological images to assist in building networks for radiological image classification may therefore enable creation of larger, more accurate networks than would be possible using radiologic images alone [8, 16].

We have found that even if a large number of radiology images are obtained, another major challenge is curation of clinical images used as input data. Although the Digital Imaging and Communications in Medicine (DICOM) format has the capacity to store images and accompanying metadata such as patient information and image modality, this information is inconsistently present and can vary by equipment manufacturer. Metadata that are obvious to a radiologist from cursory inspection of the image, such as the view orientation, are particularly inconsistent. When metadata needed to identify appropriate input is missing or inconsistent, the number of images that can reasonably be manually curated may be significantly smaller than the number of images that would otherwise be available. Tools to identify correct metadata values for large sets of images with little or no human intervention would therefore facilitate efficient use of all available image data.

### Objective

We sought to develop a deep convolutional neural network to rapidly and automatically classify view orientation of chest radiographs, to demonstrate the applicability of these techniques to classification of radiographs, and to provide highly accurate classification of large numbers of radiographs for use as input in subsequent machine learning investigations.

## Methods

### Image Collection and Training, Validation, and Test Set Creation

IRB approval was obtained for this HIPAA-compliant study. One thousand seventy-six one- and two-view chest radiographs obtained at the University of California, San Francisco (UCSF) between January 2013 and July 2015 were identified using Softek Illuminate (Version 3.5, Prairie Village, KS), and corresponding images were extracted from the PACS. Seven hundred fifty-two studies were two-view and 324 were single-view; some studies contained multiple images of the same view. In total, 1885 images from 909 patients were obtained. The images were converted to greyscale 8-bit lossy JPEG format, and min-max windowing was applied using DICOM Toolkit (DCMTK) (Version 3.6.0), which, by visual inspection, preserved enough detail for the human annotators to confidently classify the images while reducing the file sizes of the input data to reduce computational requirements and memory use.

Two authors (A.R., a hospitalist with 3 years of experience and A.T., a board-certified radiologist with 5 years of experience) independently labeled every JPEG image as frontal or lateral view; any conflicting assignments were resolved by A.T. Three images were excluded due to uninterpretable image quality.

Each image was resized to 256 by 256 pixels using ImageMagick (Version 6.7.7–10); non-square images were squashed to this size. The images were then randomly split into training, validation, and test sets with 1129, 376 and 377 images, respectively.

The training and validation sets were augmented by applying a number of image transformations: horizontal and vertical reflection, rotation by 2 or 90°, translation of 3 pixels in cardinal or ordinal directions, pixel spread (swap each pixel with a random adjacent pixel), noise reduction (replace each pixel with the value just before or after the median value in a neighborhood of 2 or 5 pixels), and random noise addition. In total, each image served as the progenitor of 106 child images with the label inherited from the parent image. This resulted in a total of 119,674 training images and 39,856 validation images.

To serve as an independent test set, we obtained a publicly available set of anonymized chest radiographs in PNG format [17]. We randomly selected 1000 of those images and manually annotated each as frontal or lateral using the procedure outlined above.

### Model Creation and Selection

Models were trained and developed using the NVIDIA Deep Learning GPU Training System (DIGITS) DevBox (Version 3), which employs the Caffe framework [18]. The results of the model were analyzed using Python (Version 2.7).

The initial model was adapted from the base GoogLeNet convolutional neural network created by Szegedy et al. [19]. This model, which we refer to as “color ImageNet,” uses 22 convolutional layers including 9 inception modules and was trained on over 1.2 million color images from the ImageNet Large Scale Visual Recognition Challenge (ILSVRC) 2015 repository [20].

Since the radiographs of interest are greyscale images, we created two additional models trained on greyscale images. For the “full-greyscale ImageNet” model, we converted all images in the ILSVRC 2015 repository to greyscale and used these to retrain the initial model. We also created a “subset-greyscale ImageNet” model by retraining color ImageNet with a greyscale subset of 100 image classes from five categories in the ILSVRC 2015 repository that we felt were most similar to our images: plants, geologic formations, natural objects, fungi, and random artifacts. The subset contained a total of 128,769 images in the training set and 5000 images in the validation set. In both cases, we retrained the initial GoogLeNet model for 30 epochs with a base learning rate of 0.0005 that was reduced tenfold every ten epochs. The parameters were optimized using a Nesterov solver with a mini-batch size of 24, momentum of 0.9, and weight decay of 0.0005. We allowed all layers to be fine-tuned. We refer to these three models as “pre-trained.”

We transferred these networks and their newly learned parameters to apply them to the training and validation images from our chest radiograph data. We retrained each of the three pre-trained models—the color ImageNet model, full-greyscale ImageNet, and subset-greyscale ImageNet model—using three different methods, resulting in nine candidate models, which we refer to as “fine-tuned” models. The three methods were fine-tuning only the fully connected layers of the model using only original radiograph images (no augmentation); fine-tuning only the fully connected layers using the augmented image sets; and fine-tuning all layers of the network using the augmented image sets. We did not attempt to fine-tune all layers of the networks using only the original radiograph training set (without augmentation) because there were too few images compared to the number of parameters that would be involved in such a retraining. For this transfer learning, a learning rate of 0.005 was used to fine-tune only fully connected layers and 0.0005 to fine-tune all layers. Stochastic gradient descent was used, with all other parameters identical to those in the previous training step.

Validation performance between models was assessed using chi-squared statistics with a significance level of 5 %. To assess the effect of pre-training and different fine-tuning, we pooled the validation accuracy of relevant models; for example, the validation performance of a pre-trained color ImageNet model was obtained by pooling the performance of the three fine-tuned models that used the model pre-trained on the color ImageNet data.

From the nine fine-tuned models, we chose a final model based primarily on validation accuracy and secondarily on the number of images used for pre-training. We then applied the network to our separate test set of 377 images and to the 1000-image test set from the publicly available data. We evaluated model speed based on time required to classify the 1377 test images. We also tested the robustness of the network on transformed versions of one frontal and one lateral test image, with modifications of text labels, rotation, obscuring half the image, and cropping.

**Fig. 1:**
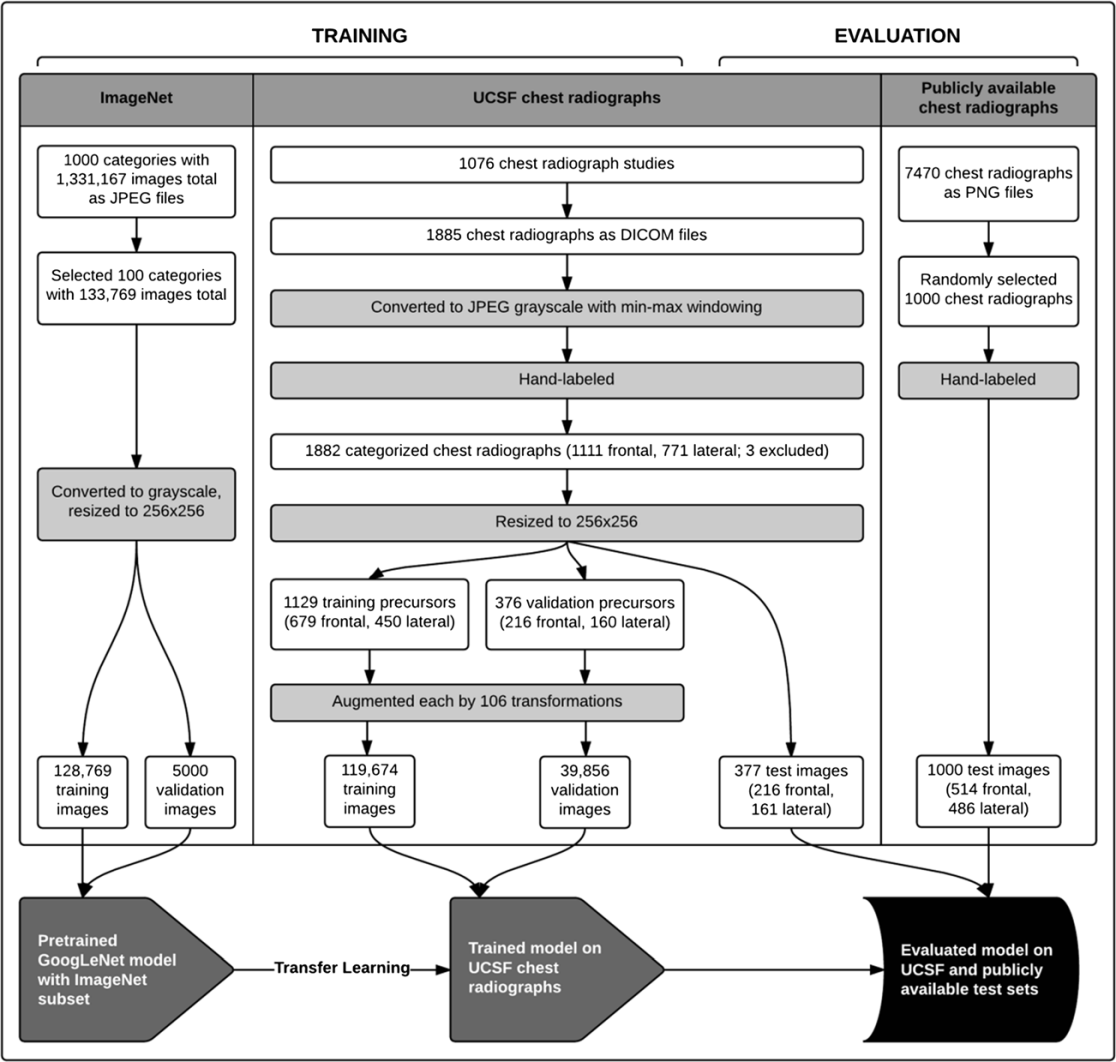
Methodology and data flow of image preparation and model creation. The flowchart outlines the pathway of image collection, selection, processing, and manual labeling (for the chest radiographs) for each image source; division of prepared images into test, training, and validation sets; and training and evaluation of the GoogLeNet model using image sets from sources as indicated in each step. *White rectangular boxes* indicate numbers of images at collection, selection, division, and post-processing steps, as well as numbers of categories (for ImageNet) and studies (for the UCSF chest radiographs) in which the images were contained. Image processing and labeling steps are in *grey rectangular boxes*

The training and evaluation process is summarized in Fig. 1.

### Model Assessment

The models returned two complementary predictions for each image: the probability of a frontal image or a lateral image. For consistency, we used the probability of an image being a frontal image (PFI) for analysis. We calculated a cutoff that maximizes the Youden Index [21] (*J*) such that images with a PFI ≥ J would be classified as a frontal image; otherwise, an image would be classified as a lateral. A range of PFI values maximized the Youden Index, and we chose the higher end of the range as the cutoff.

We used a standard two-by-two table to assess model accuracy, which we defined as the proportion of images correctly classified. 95 % confidence intervals (CI) were calculated conservatively using the Clopper and Pearson method [22].

## Results

### Model Selection

**Fig. 2:**
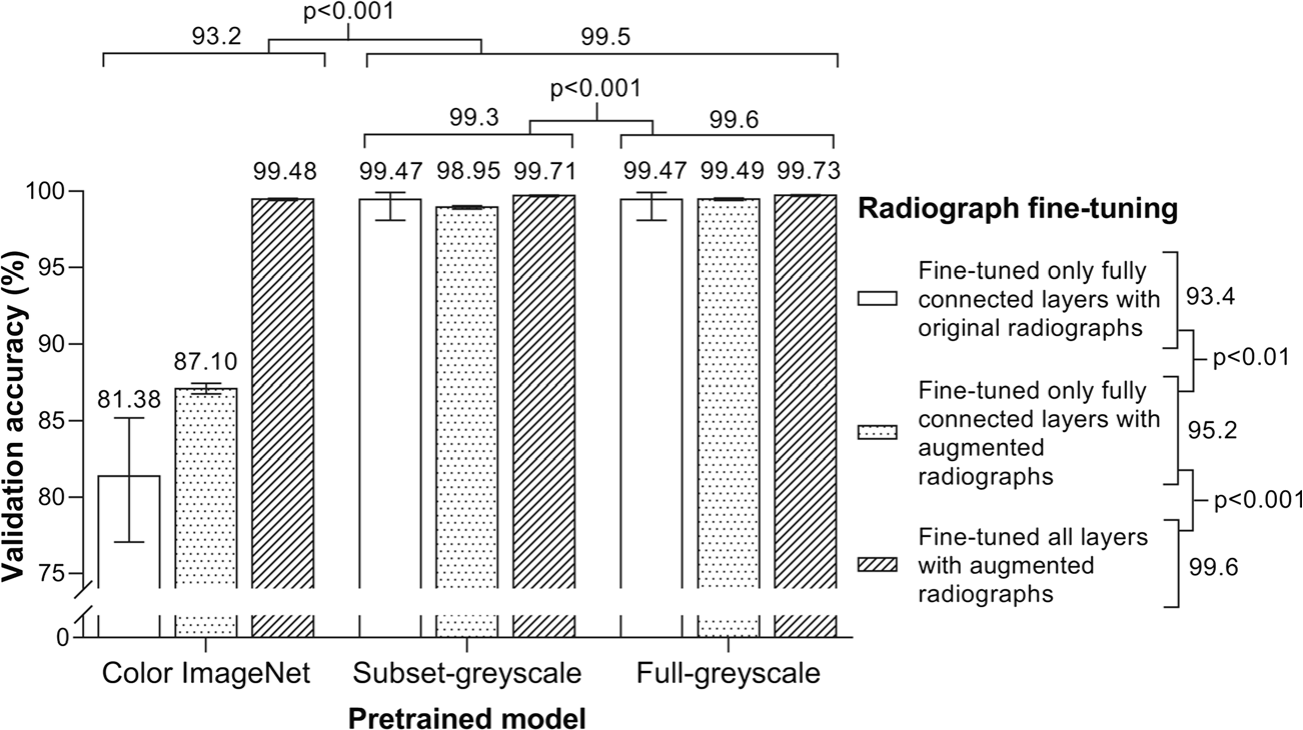
Validation accuracy for nine models. Classification accuracy, based on top prediction, of the UCSF chest radiograph validation set is graphed for the nine different models, grouped by pre-training and fine-tuning methods. *Error bars* mark 95 % confidence intervals. Labels above the graph and to the right of the legend show pooled comparisons and chi-square test results. For the models fine-tuned on original radiographs, validation sets consisted of 376 original radiographs. For models fine-tuned on augmented radiographs, validation sets consisted of 39,856 augmented radiographs

The effects of pre-training with ImageNet data and fine-tuning with augmented radiology data are shown in Fig. 2. Pre-training with greyscale ImageNet data (either full or subset) led to higher validation accuracy on chest radiographs compared to the standard models that were trained with color ImageNet data (99.5 vs 93.2, *p* < 0.001). Further, pre-training with the full-greyscale ImageNet data led to a statistically higher validation accuracy compared to pre-training with the subset-greyscale ImageNet data (99.6 vs 99.3, *p* < 0.001).

Fine-tuning the fully connected layers with the augmented training set produced better validation performance than the non-augmented (95.2 vs 93.4, *p* < 0.01). When using the augmented training set, validation accuracy was higher when all layers were fine-tuned compared to when only the fully connected layers were fine-tuned (99.6 vs 95.2, *p* < 0.001). When limited to the greyscale models, augmentation and fine-tuning of all layers led to better performance than fine-tuning of just the fully connected layers (99.7 vs 99.2, *p* < 0.001; not shown in figure).

We proceeded with the full-greyscale ImageNet model that had all layers fine-tuned with the augmented chest radiograph images, which had a validation accuracy of 99.73 % (95 % CI 99.67–99.78 %).

### Model Assessment

**Fig. 3:**
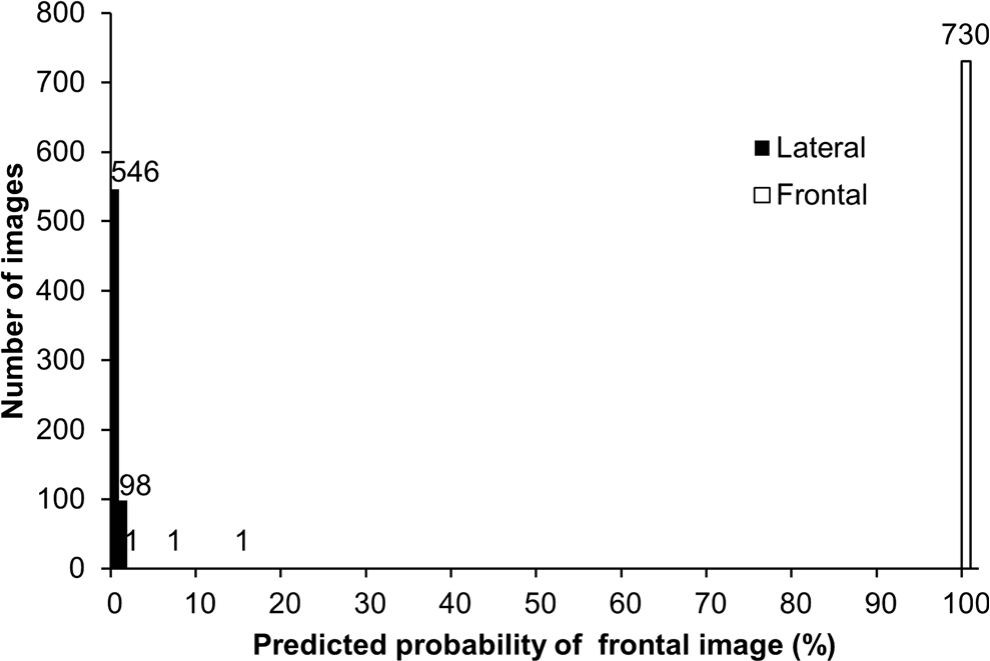
Histogram of test set classification results. Graph shows frequency of images by predicted probability of frontal images, using bins of size 1. *Shading* indicates actual class of images. Images from both the UCSF test set and publicly available set were included

**Fig. 4:**
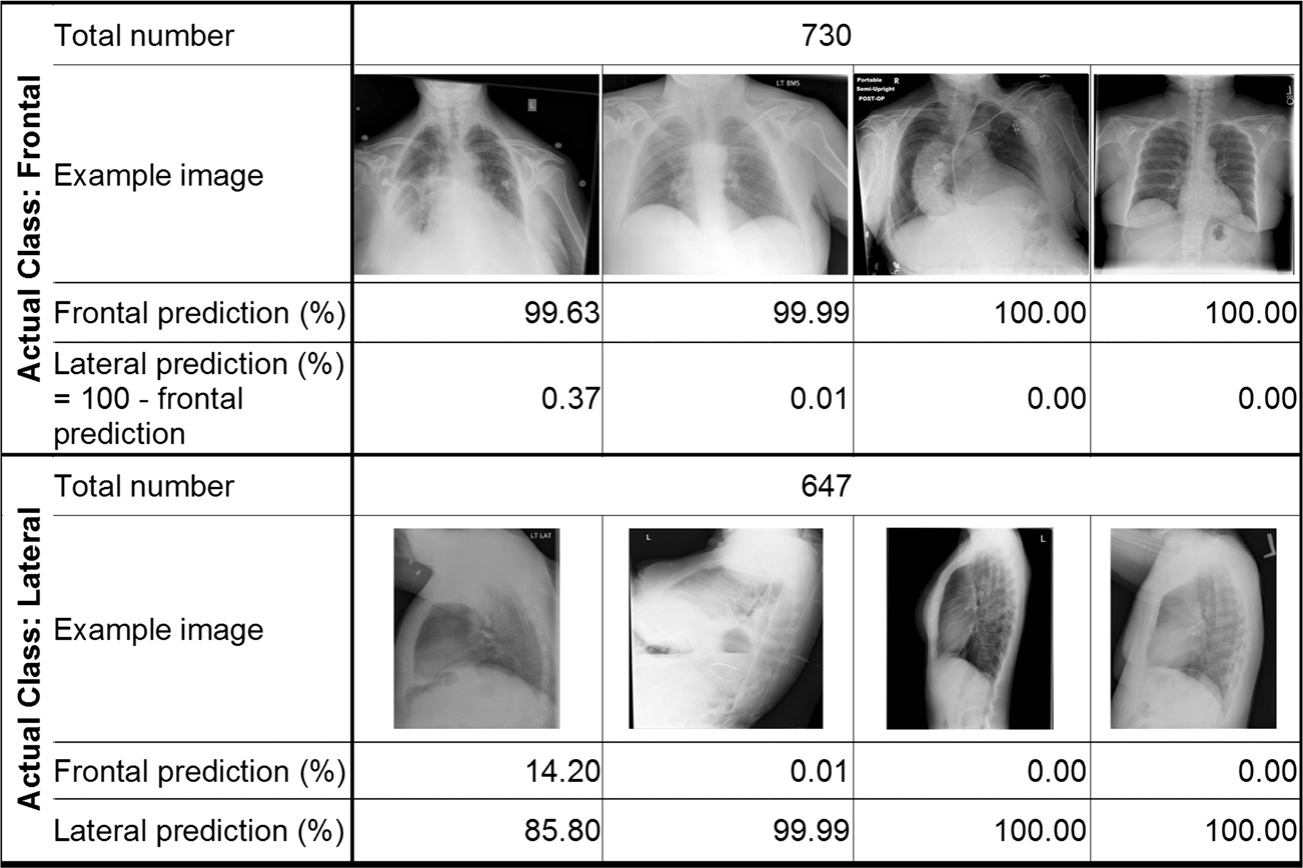
Example test images and classification results. Examples of frontal and lateral images are shown with their frontal and lateral prediction probabilities listed underneath each. Images are all from either the UCSF test set or publicly available set

Our algorithm correctly classified 100 % (95 % CI 99.73–100 %) of both the UCSF test images and the publicly available images, based on the Youden Index binary classification scheme. Figure 3 is a histogram of the PFI for the test images, which demonstrates that the network was able to separate the frontal and lateral images. There were several lateral radiographs that were less confidently predicted to be a lateral; despite this, there was still complete separation of classes. Figure 4 demonstrates examples taken from the UCSF and publicly available test sets and the classification probabilities produced by the algorithm.

**Fig. 5:**
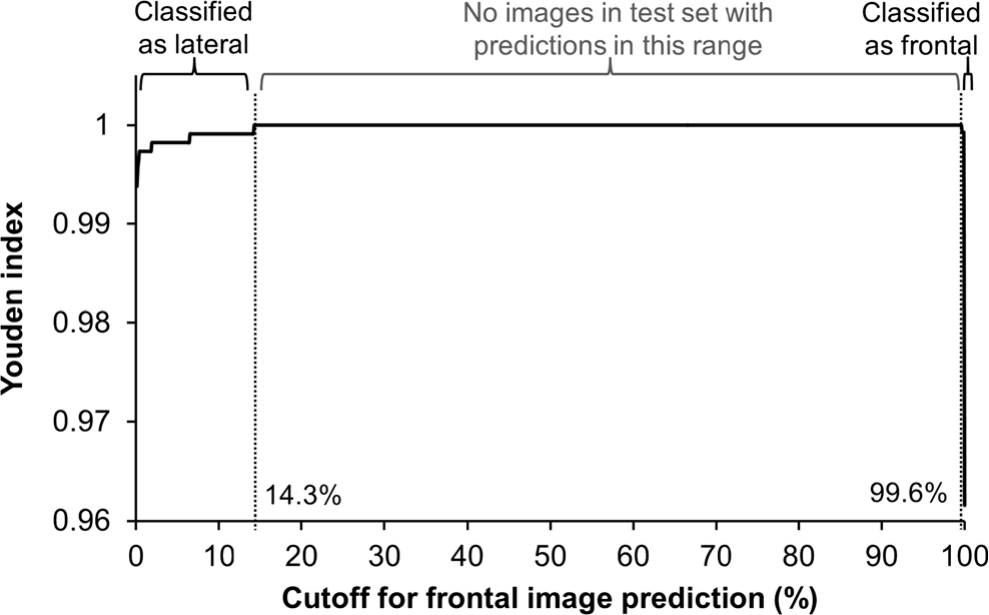
Classification cutoff determination using Youden Index. Youden Index is plotted against cutoffs determined using predicted probability of being a frontal image (PFI), incremented by 0.1 % in the range 0 to 100 %. Images from both the UCSF test set and publicly available set were included. *Labels above plot* indicate true image labels: true lateral images in the test sets all had PFI < 14.3 %, true frontal images all had PFI ≥ 99.6 %, and no test images had PFI in the intermediate range

The cutoff was set to *J* = 99.6. The Youden Index was maximized for PFI between 14.3 and 99.6 %, and no images in either test set had a PFI in this range (Fig. 5).

The model classified the test images at a rate of 38 per second.

**Fig. 6:**
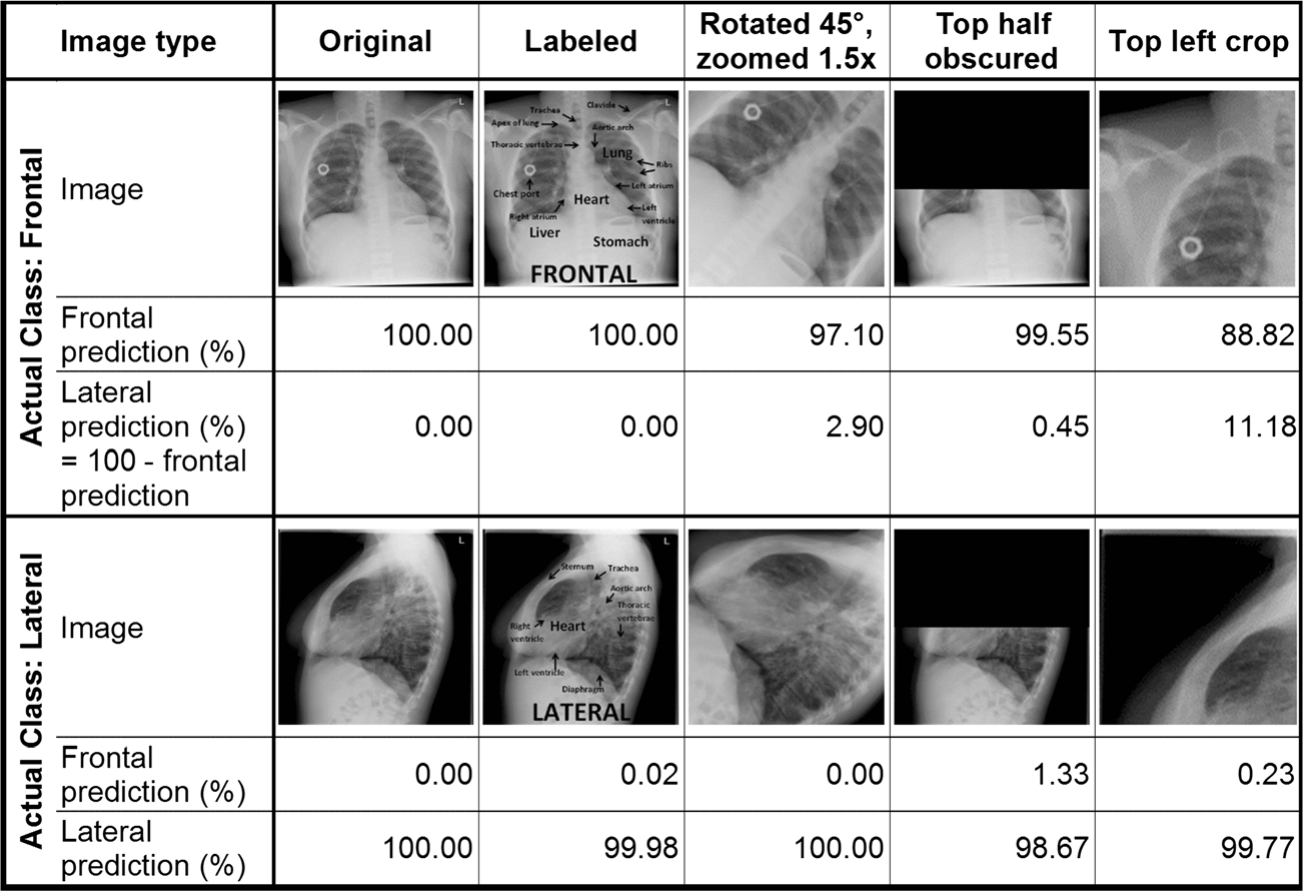
Transformed images and classification results. One frontal and one lateral image from the test sets and four transformed versions of each are shown with their frontal and lateral prediction probabilities listed underneath each. Transformations are described in the *top row*

Transformed images and their classification results are shown in Fig. 6.

## Discussion

We demonstrate that non-radiological images used in concert with a relatively small number of radiologic images, augmented through standard techniques, are effective in creating a deep learning network that performs with very high accuracy on classification of chest radiograph view orientation.

This study demonstrates that retraining of pre-existing, more general networks using greyscale images improves performance of classification of features in radiographs. Prior studies suggest that transfer learning, or using networks trained on one set of images to classify a separate set of images, is most effective when the sets are similar [16], and we hypothesized that convolutions trained to optimize classification of color images may not be as effective on greyscale images. We also demonstrate that significant improvements in pre-training with greyscale images can be achieved by using only a subset of ImageNet rather than its entirety, which reduces the time necessary to pre-train the networks.

Our report also demonstrates the effectiveness of augmenting radiology images to improve classification performance. Given the millions of parameters available to be tuned for the network, and the limited number of curated radiographs we had available for training, we augmented the training set data to reduce overfitting. Further, this training set based on a modest number of images from a single center was able to generate algorithms that generalized well, as we were able to achieve 100 % accuracy on a test set of images obtained from a different medical center.

Even without pre-training on greyscale images, a network pre-trained on color images can achieve high accuracy through training on an augmented set generated from a limited number of radiology images. However, we show that this performance is improved when all layers are allowed to fine-tune using the augmented images rather than just the fully connected layers, which we suspect is due to a better deep representation of the images.

Our work extends prior studies on image classification applied to chest radiography, and for a new application. Boone et al. [23] used a neural net to make predictions about image rotation and flipping of chest X-rays by making inferences about features such as the location of the mediastinum. The algorithm evaluated column and row vectors of summed pixels from the images, rather than classifying the entire raw image, a reasonable approach given the computational power available at the time. The goal of this work was to automate the task of correctly orienting scanned film images that were converted to digital images at the beginning of the PACS era. Although the application for the algorithm was different, the underlying challenge was the same: the need for a method to minimize human curation of a very large image dataset with incomplete and often unreliable metadata. Other groups have recently used explicit image analysis and anthropometric algorithms to handle chest X-ray view classification [24]. Our approach requires minimal pre-processing of images and operates on an open-source software stack that is robust, easy to implement, and has higher accuracy. Other recent uses of recurrent neural networks to caption chest radiographs have assumed a dataset with correct and pre-defined metadata, which we have not found in our clinical database [15], or have used orders of magnitude fewer images than our work [8].

This study was limited in scope given the relatively simple task of classifying orientation of chest radiograph images as frontal or lateral. This task does not require detection of subtle abnormalities, such as a pneumothorax, so the ability of deep learning to identify pathology has not yet been proven. However, an end-to-end solution requires safeguards that the network is fed image data appropriate for the classification task at hand, so ensuring the correct metadata is a critical first task. It is not widely appreciated that large repositories of images may have incorrect metadata that may preclude or confound creation of more sophisticated algorithms. Moreover, we hand-labeled images rather than extracted labels automatically from radiology reports, which implies an important role for natural language parsing in future refinements of our image processing workflow.

## Conclusion

In summary, we report a method to automate metadata annotation using deep learning methodology. We also demonstrate the effectiveness of network pre-training using non-radiology images and of augmentation of radiology images to achieve high-fidelity classification and generalizable accuracy. This is proof of concept that deep learning, which has revolutionized computer vision research, may drive advances in analysis of radiologic images.
